# Simulation-based education in paramedic training: effects of perceived competence and practice frequency on clinical skills

**DOI:** 10.1186/s12909-025-08541-5

**Published:** 2026-02-18

**Authors:** Demet Turan, Hilal Pekmezci

**Affiliations:** 1 Recep Tayyip Erdogan University, Rize, Turkey; 2 Health Services Vocational School, Recep Tayyip Erdogan University, Rize, 61080 Turkey

**Keywords:** Paramedic education, Clinical skills, Perceived competence, Practice frequency, Simulation-Based education, Self-Efficacy

## Abstract

**Background:**

Simulation-based education is increasingly used in health professions training to strengthen clinical competence and self-efficacy. Paramedic students, in particular, need repeated practice opportunities to acquire essential intervention skills. Limited exposure to clinical scenarios may reduce their perceived competence and procedural success. This study aimed to evaluate the effectiveness of simulation-based training in improving paramedic students’ clinical skills and to examine the impact of perceived competence and practice frequency on performance outcomes.

**Methods:**

A single-center, one-group pretest–posttest quasi-experimental design was employed with 60 students enrolled in the Paramedic Program of a public university in Türkiye. Data were collected using researcher-developed forms measuring demographics, training-related evaluations, perceived competence, and frequency of practice, with these variables assessed using Likert-type items. Eight procedural skills were assessed through structured observation based on standardized guidelines. Statistical analyses included descriptive statistics, paired samples t-tests, Wilcoxon signed-rank tests, and Spearman correlation analysis.

**Results:**

Before training, most students had limited or no experience with core interventions, and their success rates were low. Following simulation-based education, statistically significant improvements were observed in all skills (*p* < 0.001), with a very large effect size for the increase in practice frequency (Cohen’s *d* = 2.94). A 100% success rate was achieved in oxygen administration and fracture stabilization, with notable gains in advanced procedures such as intubation (90%), life support (81.7%), defibrillation (76.7%), and cardiac arrest management (75%). Perceived competence scores increased significantly (*p* < 0.001). A positive correlation was found between frequency of practice and perceived competence, with a correlation coefficient of Spearman’s *r* = 0.303 (*p* = 0.019).

**Conclusions:**

Simulation-based education is effective in enhancing paramedic students’ clinical performance, practice frequency, and self-perceived competence. These findings provide practical guidance for curriculum planners by supporting the structured integration of high-fidelity simulation, repeated practice opportunities, and simulation-based assessment methods into paramedic education programs. However, given the single-group pretest–posttest design, the findings should be interpreted with caution regarding causal inference.

**Trial registration:**

Not applicable.

## Introduction

Paramedic education aims to equip students with the skills necessary to perform rapid, effective, and safe interventions in prehospital critical situations [1]. Achieving these objectives requires more than theoretical knowledge. Practice-based learning, particularly when supported by high-fidelity simulation, significantly enhances technical proficiency as well as communication skills during critical interventions.

Comprehensive systematic reviews conducted between 2010 and 2021 have demonstrated that critical procedures such as patient assessment, treatment processes, and especially airway management lead to significant improvements in both perceived competence and objective performance [1, 2]. For example, Bienstock et al. (2022) reported that high-fidelity simulations enhanced both performance and perceived competence in airway management [1]. Similarly, Nielsen et al. (2021) found that simulation-based team training had positive effects on preparedness for airway management and clinical performance [3].

There is a strong relationship between the frequency of students’ hands-on practice and their perceived competence. In particular, repeated experiences with life-saving interventions such as endotracheal intubation, cardiopulmonary resuscitation (CPR), and defibrillation have been shown to substantially improve both subjective perceptions of competence and objective clinical skills [1, 4]. The literature also emphasizes a direct link between practice frequency and self-efficacy.

Practice experiences gained during life-threatening interventions not only enhance perceived competence but also lead to measurable performance outcomes [4]. This improvement is associated not only with the number of practices performed but also with their contextual appropriateness and repeatability. Training delivered through mobile simulation laboratories, in-ambulance modules, and high-fidelity simulation centers enables students to engage with realistic and diverse clinical scenarios, thereby supporting the effective transfer of theoretical knowledge into practical competence [1, 5, 6].

Simulation-supported education methods have also been found effective in enhancing both professional competence and self-efficacy among paramedic students. Studies conducted in Türkiye have shown that when opportunities for practice in essential procedures such as childbirth management, bleeding control, and fracture stabilization are limited, students tend to report lower perceptions of competence [7, 8]. These findings highlight the need for educational programs to expand opportunities for hands-on learning. Simulation-based education has proven effective in enhancing clinical skills and learner confidence in healthcare education. High-fidelity simulation, particularly in nursing education, has been shown to significantly improve students’ self-confidence, satisfaction, and performance in critical areas such as mental health, airway management, and pediatric care [9–12]. These findings are consistent with global evidence showing that simulation-based training improves clinical competence and self-efficacy across various healthcare fields [13, 14].

In this study, perceived competence refers to students’ self-assessment of their ability to perform specific clinical skills, while practice frequency denotes the number of times students actively performed clinical interventions during training. From a theoretical perspective, Bandura’s self-efficacy theory provides a strong framework for understanding the relationship between practice frequency, perceived competence, and skill acquisition. Self-efficacy refers to individuals’ beliefs in their capacity to organize and execute the actions required to manage prospective situations [15]. In health professions education, repeated mastery experiences such as performing clinical procedures in simulated environments are considered the most powerful source of self-efficacy. As students repeatedly practice critical interventions in realistic simulation settings, their confidence increases. This increase in confidence positively influences motivation, persistence, and actual performance. Therefore, simulation-based education may enhance clinical skill performance indirectly by strengthening students’ self-efficacy through structured and repeated practice opportunities. However, few studies have examined the impact of simulation-based education on both perceived competence and objectively assessed clinical skills among paramedic students in Türkiye.

This study aimed to evaluate the effectiveness of simulation-based education in improving paramedic students’ clinical skills and to examine the relationship between perceived competence, practice frequency, and performance.

## Methods

### Study design

This study was conducted using a quasi-experimental one-group pretest–posttest design. The effectiveness of the training intervention was evaluated by comparing paramedic students’ clinical skill performance before and after the intervention.

### Population and sample

The population of this study consisted of students enrolled in the Paramedic Program at a public university’s vocational school of health services during the fall semester of the 2022–2023 academic year, who continued their education throughout four academic semesters. Of the 66 students registered in the program during the relevant period, 6 students were excluded from the study due to transferring to another program, failure to renew enrollment, or withdrawal due to health issues. As a result, the study was completed with a total of 60 participants.

All eligible students enrolled during the study period were included; therefore, the study was conducted using a census sample.

The population of this study consisted of students enrolled in the Paramedic Program at a public university’s vocational school of health services during the fall semester of the 2022–2023 academic year, who continued their education throughout four academic semesters. Of the 66 students registered in the program during the relevant period, 6 students were excluded from the study due to various reasons such as transferring to another program, failure to renew enrollment, or withdrawal due to health issues. As a result, the study was completed with a total of 60 participants.

All eligible students enrolled during the study period were included, and the study was therefore conducted using a census sample. Because the sample size was determined by population availability rather than statistical considerations, no a priori sample size calculation was used to guide recruitment, and the study was framed as exploratory. To provide context regarding sample adequacy, conventional power considerations for paired comparisons suggest that a medium-sized effect could be detected with fewer participants than were included in the present study.

### Inclusion criteria

- Being enrolled in the relevant higher education program during the fall semester of the 2022–2023 academic year.
- Having adequate verbal communication skills.
- No history of psychiatric illness (assessed based on students’ self-report).
- Voluntary agreement to participate in the study.

**Fig. 1:**
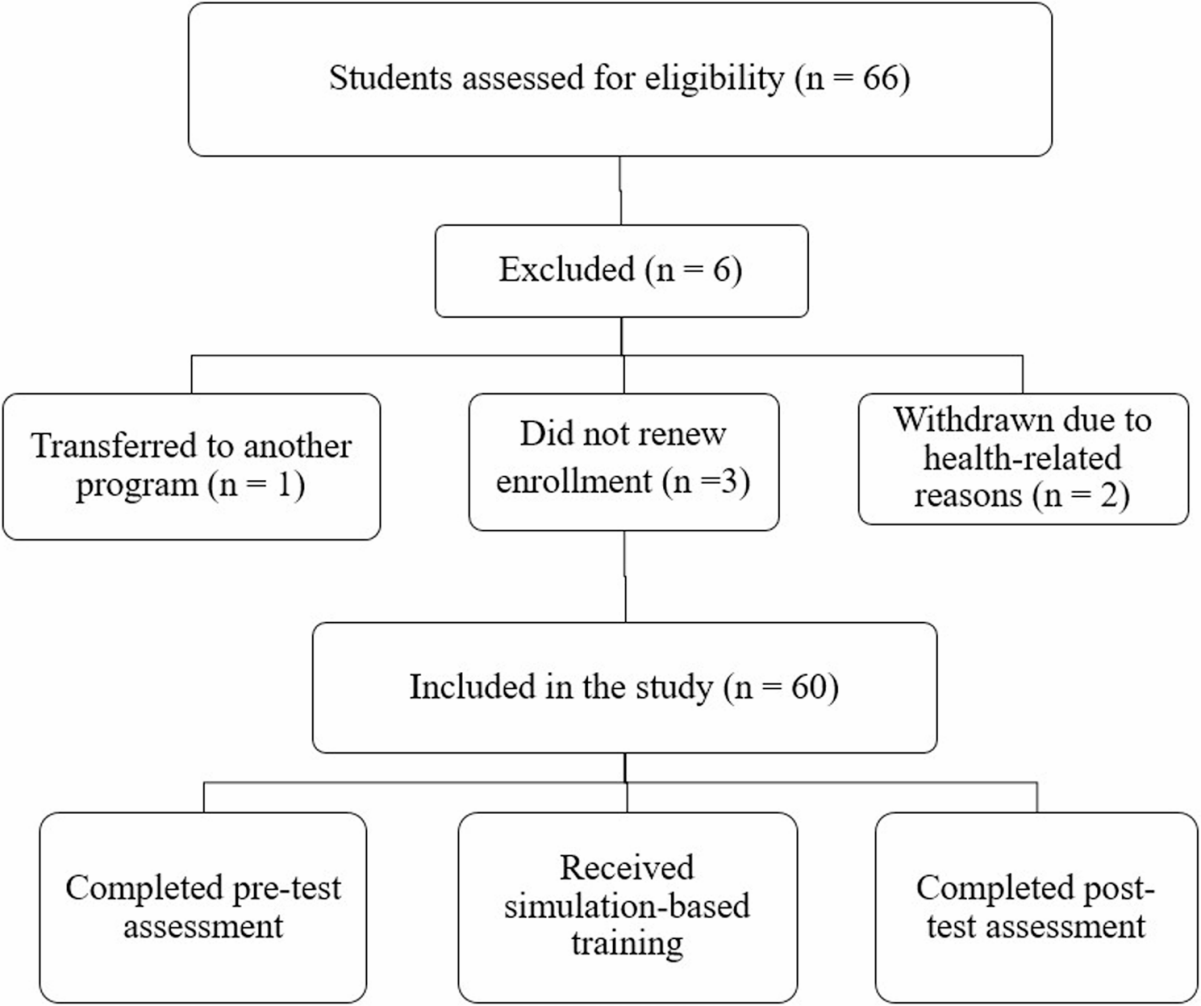
Flow diagram of participant inclusion and exclusion

The flow diagram illustrating the inclusion and exclusion of participants in the study is presented in Fig. 1.

### Data collection tools

Three different instruments were used to collect data in this study: the *“Personal Information Form*,” the “*Training Evaluation Form*,” and the “*Clinical Skill Assessment Form*” Data were collected at two points: as a pretest at the beginning of the training and as a posttest before graduation.

### Personal information form

This form was developed by the researchers based on a review of the relevant literature and includes questions related to the students’ demographic characteristics such as gender, age, height, weight, place of residence, and type of high school graduated [4, 16].

### Training evaluation form

This form was developed based on a comprehensive review of the relevant literature on simulation-based education and paramedic training, including both international and national studies [1, 3, 2, 7, 8]. It consists of three sections and a total of 44 items.

#### Section 1

The first section includes 17 items aimed at assessing whether students received training on topics included in the curriculum. Participants rated their level of training on a 5-point Likert scale ranging from 1 (Not trained at all) to 5 (Extensively trained). The Cronbach’s alpha reliability coefficient for this section was calculated as 0.889 in this study [4, 16].

#### Section 2

The second section contains 12 items evaluating the frequency with which students performed various medical interventions. The frequency of practice was rated on a four-point scale: Never practiced, Practiced once, Practiced 2–5 times, and Practiced 6 or more times. The Cronbach’s alpha coefficient for this section was found to be 0.782 [4, 16].

#### Section 3

The third section includes 15 items measuring students’ perceived competence in performing specific medical procedures. Responses were recorded using a 5-point Likert scale ranging from 1 (Strongly Disagree) to 5 (Strongly Agree). The Cronbach’s alpha coefficient for this section was calculated as 0.922 [4, 16].

### Clinical skill assessment form

This form was developed to evaluate students’ practical skills and was used to measure their level of success in performing specific clinical interventions. A total of eight core procedural skills were assessed through direct observation by relevant faculty members. Each procedure was structured according to standardized steps derived from the guidelines of the American Heart Association [17] and the American College of Surgeons [18].

A four-level scoring system was applied for each skill as follows:

1. Unsuccessful
2. Partially successful
3. Successful on second attempt
4. Successful (correct and complete on first attempt)

The maximum score a student could receive for each skill was 4, with a total possible score of 32 across all eight procedures. For the purpose of reporting skill-specific success rates, performances rated as “successful on the second attempt” (score 3) and “successful” (score 4) were considered successful for each individual procedure. Success rates were calculated as the proportion of students meeting these criteria for each skill. This form was used to objectively measure students’ competency in practice-based clinical skills. To ensure scoring consistency, all assessments were conducted using standardized checklists and predefined scoring criteria derived from international clinical guidelines.

Although two instructors were present during the clinical skill assessment sessions, all performances were scored by a single instructor using standardized checklists and predefined scoring criteria to ensure consistency across evaluations. Therefore, inter-rater reliability was not calculated. The use of a single rater may introduce potential rater bias, which should be considered when interpreting the results.

### Data collection process

The data collection process was carried out between September 19, 2022, and June 20, 2024, corresponding to the full duration of the two-year paramedic education program. At the beginning of the first academic semester, prior to formal instruction, participants were administered a pretest including the Personal Information Form, the Training Evaluation Form, and the Clinical Skills Assessment Form. Upon completion of the four academic semesters and immediately prior to graduation, posttests were conducted using the same instruments. During the evaluation process, completion of the self-report forms required approximately 10 min, and the clinical skill assessment for each student was completed in approximately 30 min. It should be noted that students were exposed to multiple learning experiences throughout the program, including clinical placements and non-simulation courses. Therefore, the observed outcomes reflect the cumulative educational experience within the curriculum, with simulation-based education representing a key instructional component.

### Curriculum and weekly training structure

Each academic semester lasted 16 weeks, during which students participated in theoretical and practical training as part of the core curriculum of the paramedic program. The curriculum places strong emphasis on practice-oriented courses. In particular, Emergency Patient Care I–II–III, Professional Practice I–II, and Trauma courses include structured hands-on sessions.

Throughout the educational program, students completed approximately 992 h of theoretical instruction and 704 h of practical training. Practical education was delivered through laboratory-based sessions, in-ambulance training, and simulation activities integrated into the curriculum. This structured, practice-oriented training model was designed to enhance students’ clinical competence.

### Simulation-based training environment

Simulation activities were conducted at the university’s Clinical Simulation Training Center. During the training process, not only high-fidelity manikins (e.g., Laerdal SimMan Essential) were utilized, but also advanced virtual patient applications were employed. In particular, the BodyInteract™ software provided students with the opportunity to enhance their decision-making, intervention planning, and outcome evaluation skills within virtual clinical scenarios.

The OSCE (Objective Structured Clinical Examination) rooms within the simulation center were structured to offer practical training opportunities on critical areas such as communication with simulated/standardized patients, teamwork, motivation, and patient and staff safety.

The educational process was not limited to the laboratory setting; it was further supported by direct clinical practice in the emergency medicine units of the university’s training and research hospital. This integrated approach enabled students to gain real-world experience and effectively transfer the skills acquired through simulation into clinical practice.

Overall, the simulation-based training was conducted using a high-fidelity simulation approach. To ensure standardization across all sessions, the same simulation environment, high-fidelity manikins, virtual patient software, scenario scripts, session duration, and instructors were used for all participants. Simulation scenarios were implemented in accordance with predefined learning objectives and a standardized scenario flow and were not freely repeated outside the scheduled simulation sessions.

Debriefing was conducted immediately after each simulation session using a structured, instructor-led approach. Students’ performances were reviewed step by step based on standardized checklists, and each clinical skill was evaluated using a four-level performance classification (unsuccessful, partially successful, successful on the second attempt, and successful). Following the assessment, procedural steps and clinical decision-making processes were discussed with the students to support reflection and learning.

### Scenario application and performance monitoring

In each session, a specific clinical scenario (cardiac arrest, endotracheal intubation, trauma patient management, etc.) was implemented, and students assumed designated roles to perform scenario-based interventions. The scenarios were structured in accordance with the guidelines of the American Heart Association [17] and the American College of Surgeons [18].

During the simulations, students were individually assessed, followed by structured feedback delivered by the instructors. To monitor students’ skill development throughout the training process, individual performance was recorded on observation forms based on the procedural steps for each scenario.

At the end of the training program, post-tests were administered using the same instruments to evaluate the participants’ progress. The simulation sessions were conducted in small groups, and one faculty member observed and assessed student performance during each session. Structured feedback was provided both during and after the practice sessions, thereby reinforcing the learning process.

### Data collection procedure

The Clinical Skills Assessment Form was completed by faculty members experienced in the field. During the evaluation process, clinical skill assessments were conducted by two faculty members working simultaneously. While one instructor administered and supervised the clinical skill performance, the second instructor was responsible for scoring student performance using the standardized Clinical Skill Assessment Form. Thus, all clinical skills were scored by a single evaluator, while the second instructor supported the assessment process by conducting the skill examination. Both the pre-test and post-test assessments of the students were conducted by the same instructors, and blinding was not applied during the evaluation. However, to minimize subjectivity in scoring, standardized scoring criteria and structured observation forms were used. Both the pre-test and post-test assessments were conducted at the university’s Clinical Simulation Training Center, and consistent physical conditions (laboratory setup, equipment, simulation manikins, and software infrastructure) were maintained to control environmental variables during the assessments.

### Data analysis

The research data were analyzed using the SPSS for Windows 26.0 statistical software. The distributions related to participants’ demographic characteristics and application performance were presented using descriptive statistics (number, percentage, mean, standard deviation, median). The normality of the data distribution was assessed using the Shapiro-Wilk test. For variables that did not meet the normality assumption, the Wilcoxon Signed-Rank Test was used, while the paired samples t-test was applied to variables with a normal distribution.

These tests were used to compare dependent groups in pre- and post-training variables. In addition, frequency and percentage distributions for categorical data such as application success and perceived competence were calculated and presented in tables. Cohen’s d effect size was calculated to determine the magnitude of significant differences observed in the analyses. A significance level of *p* < 0.05 was considered for all statistical tests.

An achieved power analysis was conducted to assess the adequacy of the sample size for the primary paired-samples t-test, indicating sufficient statistical power (power > 0.99).

### Ethical considerations

The students were informed that participation in the study was entirely voluntary, and only those who agreed to participate voluntarily were included in the research. Since the study involved human subjects, the principle of “Informed Consent” was fulfilled as an ethical requirement to ensure the protection of individual rights. Additionally, the principle of “Respect for Human Dignity” was also taken into consideration. Participants were informed that the information obtained about them would not be disclosed to others, and the “Principle of Confidentiality” was strictly observed. The study was conducted with the necessary approval obtained from the Ethics Committee for Social and Human Sciences of the relevant university (Ethics Committee No: 2022/220).

### Clinical trial number

Not applicable.

## Results

When the demographic characteristics of the participants were examined, it was found that 83.3% of them were female, with a mean age of 21.53 ± 2.39 years. The mean body weight was 64.12 ± 10.72 kg, and the mean height was 165.42 ± 8.072 cm. In terms of residence, 75% of the participants were staying in government dormitories. Regarding educational background, 40% were graduates of health vocational high schools, while 60% had graduated from other types of high schools (Table 1).

**Table 1:** Descriptive characteristics of the participants

		Mean ± S.D	Min-Max	*n*	%
Gender	Male			10	16.7
	Female			50	83.3
Age		21.53 ± 2.39	19–32		
Body weight (kg)		64.12 ± 10.72	45–98		
Height (cm)		165.42 ± 8.072	152–188		
Place of Residence	Goverment Dormitory			45	75
	With Family			11	18.3
	Student House			4	6.7
Type of High School Graduated	Health Vocational High School			24	40
	General High Schools			36	60

The percentages of students who had never performed specific medical interventions before and after the training are presented in Table 2. Prior to the training, the majority of students reported no prior experience with several interventions. For example, the proportion of students who had never performed endotracheal intubation was 98.3% before the training, which decreased to 31.7% after the training. Similarly, the percentage of students who had never practiced childbirth management decreased from 96.7% to 25.0%, and the proportion of those who had never used a defibrillator decreased from 96.7% to 21.7%.

**Table 2:** Percentage of students who had never performed medical interventions before and after the training

Medical Intervention	Pre % No Practice	Post % No Practice
Cardiopulmonary resuscitation (CPR)	93.3%	11.7%
Artificial respiration	86.7%	45.0%
Use of defibrillator	96.7%	21.7%
Intravenous (IV) line insertion	73.3%	1.7%
Oxygen administration via mask/Ambu	85.0%	3.3%
Endotracheal intubation	98.3%	31.7%
Bleeding control	88.3%	15.0%
Fracture stabilization	93.3%	18.3%
Use of ambulance equipment	88.3%	3.3%
Childbirth practice	96.7%	25.0%
Emergency care in cardiac arrest (case-based)	96.7%	6.7%
Emergency drug/fluid administration	98.3%	28.3%

Changes in the frequency scores of medical interventions before and after the training are presented in Table 3. When examining the mean and median scores, it is evident that the frequency of performing all assessed interventions increased following the training.

**Table 3:** Pre- and Post-Training mean and median scores of medical interventions

Medical Intervention	Pre Mean ± SD	Post Mean ± SD	Pre Median	Post Median
Cardiopulmonary resuscitation (CPR)	1.12 ± 0.49	2.75 ± 0.88	1.00	3.00
Artificial respiration	1.18 ± 0.50	1.93 ± 0.99	1.00	2.00
Use of defibrillator	1.05 ± 0.29	2.25 ± 0.82	1.00	2.00
Intravenous (IV) line insertion	1.55 ± 1.03	3.95 ± 0.39	1.00	4.00
Oxygen administration via mask/Ambu	1.32 ± 0.85	3.47 ± 0.77	1.00	4.00
Endotracheal intubation	1.02 ± 0.13	2.17 ± 0.94	1.00	2.00
Bleeding control	1.22 ± 0.67	2.57 ± 0.96	1.00	3.00
Fracture stabilization	1.10 ± 0.44	2.47 ± 0.91	1.00	3.00
Use of ambulance equipment	1.18 ± 0.57	3.22 ± 0.80	1.00	3.00
Childbirth practice	1.07 ± 0.41	2.05 ± 0.79	1.00	2.00
Emergency care in cardiac arrest (case-based)	1.05 ± 0.29	2.95 ± 0.79	1.00	3.00
Emergency drug/fluid administration	1.03 ± 0.26	2.50 ± 1.11	1.00	3.00

The results presented in Table 4 indicate a statistically significant increase in students’ mean scores for medical intervention practice in the post-training period. The mean score before the training was calculated as 1.16 ± 0.36, while the post-training mean was 2.69 ± 0.50. The normality of the data distribution was assessed using the Shapiro-Wilk test, and the distribution was found to be normal (W = 0.98, *p* = 0.528). The paired samples t-test revealed a significant difference between the pre- and post-training measurements (t(59) = -22.80, *p* < 0.001). Furthermore, the magnitude of this difference was evaluated as a very large effect size, with Cohen’s d = 2.94.These results demonstrate that students had more opportunities to perform various essential and life-saving medical interventions during the training process, highlighting the effectiveness of the practice-oriented educational approach.

**Table 4:** Paired samples t-Test results for the comparison of the number of medical interventions performed before and after the training

	Pre-Training (M ± SD)	Post-Training (M ± SD)	*t*	*p*	*d* (effect size)
Number of medical interventions	1.16 ± 0.36	2.69 ± 0.50	-22.80	< 0.001	2.94

Table 5 shows a significant improvement in students’ perceived competence across all skill areas following the training. Wilcoxon signed-rank test results revealed that post-training median scores were significantly higher than pre-training scores for all items (*p* < 0.001).

**Table 5:** Changes in students’ perceived competence scores before and after training

Skill Area	Pre Median (IQR) (25th-75th)	Post Median (IQR) (25th-75th)	z	*p*-value
I can manage a trauma patient	1 (1–1)	5 (5–5)	−7.308	< 0.001
I can perform triage	1 (1–1)	5 (5–5)	−7.270	< 0.001
I can maintain airway patency	1 (1–1)	5 (5–5)	−7.202	< 0.001
I can establish intravenous (IV) access	1 (1–1)	5 (5–5)	−7.206	< 0.001
I can perform effective CPR	1 (1–1)	5 (5–5)	−7.274	< 0.001
I can administer oxygen	1 (1–1)	5 (5–5)	−7.278	< 0.001
I can perform endotracheal intubation	1 (1–1)	5 (5–5)	−7.306	< 0.001
I can control bleeding	1 (1–1)	5 (5–5)	−7.171	< 0.001
I can transport a patient using proper lifting/carrying techniques	1 (1–1)	5 (5–5)	−7.306	< 0.001
I can stabilize fractures	1 (1–1)	5 (5–5)	−7.345	< 0.001
I can provide basic and advanced life support	1 (1–1)	5 (5–5)	−7.239	< 0.001
I can use a defibrillator	1 (1–1)	5 (5–5)	−7.235	< 0.001
I can prepare a patient for transfer	1 (1–1)	5 (5–5)	−7.236	< 0.001
I can use ambulance equipment	1 (1–1)	5 (5–5)	−7.474	< 0.001
I can manage childbirth	1 (1–1)	5 (5–5)	−7.202	< 0.001

In addition, a statistically significant positive correlation was found between post-training practice frequency and perceived competence (Spearman’s *r* = 0.303, *p* = 0.019), indicating that students who engaged in more frequent clinical practice reported higher levels of perceived competence.

To evaluate the significance of the difference between pre- and post-training perceived competence scores, the Wilcoxon signed-rank test was applied. The normality assumption was assessed using the Shapiro-Wilk test, and since the data did not follow a normal distribution (W = 0.71, *p* < 0.001), a non-parametric analysis was preferred.

The results revealed a statistically significant difference between the two measurements (V = 0.00, z = -6.81, *p* < 0.001). This finding indicates that the training process significantly improved students’ perceptions of professional competence. The relevant results are presented in Table 6.

**Table 6:** Wilcoxon Signed-Rank test results for perceived competence before and after the training

	V	z	*p*-value	Median (Pre-Training)	Median (Post-Training)
Perceived Competence Scores	0.00	-6.81	< 0.001	1.00	5.00

Table 7 presents a comparison of paramedic students’ practical skill performance before and after the training. Compared with the pre-training assessment, post-training total performance scores and success rates increased across all eight practical skills. The most pronounced improvements were observed in advanced procedures such as endotracheal intubation, basic and advanced life support, defibrillation, and cardiac arrest management, indicating a substantial enhancement in students’ clinical skill performance following the training.

**Table 7:** Comparison of total performance scores and success rates of students’ practical skills before and after the training

Skill Area	Total Mean Score (Pre)	Total Mean Score (Post)	Pre-Training Success (%)	Post-Training Success (%)
Maintaining airway patency	10.93	30.64	34.25	91.67
Oxygen administration	9.87	32.0	30.75	100.0
Endotracheal intubation	8.13	27.04	25.5	90.0
Basic and advanced life support	8.93	29.44	28.0	81.67
Defibrillation	9.33	24.64	29.25	76.67
Cardiac arrest management	8.67	25.44	27.0	75.0
Fracture stabilization	9.33	32.0	29.25	100.0
Transporting the injured with proper lifting techniques	9.20	31.04	28.75	88.33

## Discussion

The results of our study indicate that students’ hands-on experience with critical interventions increased significantly, shifting from “no prior practice” to performing these procedures more frequently (Table 2). This improvement was supported not only by simulation training but also by complementary clinical practice in hospital settings. In line with previous studies, Donath et al. (2025) demonstrated that following pediatric simulation training, endotracheal intubation (ETI) times were reduced and interruptions in chest compressions significantly decreased due to stronger intrateam coordination [19]. Similarly, a systematic review and network meta-analysis by Ando et al. (2022) reported that simulation-based training in airway management significantly enhanced behavioral performance compared to both non-simulation methods and no-intervention control groups. The same analysis also showed improvements in knowledge acquisition. These findings suggest that the reduction in “never performed” rates observed in our study reflects not only a quantitative increase but also a qualitative improvement in clinical competence [14]. A recent meta-analysis further demonstrated that simulation significantly supports the development of both technical knowledge and practical skills [20]. This result is consistent with the findings of our study.

Evidence from other contexts also reinforces this conclusion. In Nepal, a simulation-based emergency airway management program for medical interns was associated with significant improvements in knowledge, procedural competence, and self-efficacy (“I can do it”) [21]. Taken together, these findings indicate that repeated hands-on practice and simulation-based training play a key role in achieving meaningful improvements in clinical competence.

In our study, the significant increase in the number of medical interventions performed after training (Table 3) underscores the potential impact of simulation-based practice in this area. This finding is consistent with the study by Alshibani et al. (2025), which reported improvements in clinical performance, procedural success, and error awareness following a 12-month structured simulation program [22]. Similarly, a study from Norway found that air ambulance teams engaged in simulation training more frequently than ground teams and benefited more from structured assessment processes aimed at developing non-technical skills [23]. However, given the single-center setting and the absence of a control group, these findings should be considered within the scope of the present study design.

The paired samples t-test results in Table 4 demonstrate a statistically significant increase in students’ overall educational exposure following the training, with a very large effect size (Cohen’s d = 2.94). This finding suggests a strong educational impact of the simulation-based program in the present study. This result is consistent with the systematic review by Altayyari et al. (2025), which reported that simulation-based training significantly enhances paramedics’ educational engagement, procedural learning, and clinical readiness, particularly when repeated exposure and structured debriefing are used [24].

Notably, following simulation-based training, students in our study reported reaching the level of “strongly agree” in their ability to perform critical emergency interventions such as bleeding control, triage, airway maintenance, intravenous access, oxygen administration, effective CPR, and proper patient transport techniques (Table 5). This finding suggests that simulation strengthens not only technical performance but also students’ self-perceived competence. This finding is well supported by contemporary literature. El Ougli et al. (2024) reported that high-fidelity simulation-based adult CPR training not only reduced anxiety but also significantly increased self-efficacy, satisfaction, and confidence among nursing students in Morocco [25]. Similarly, in a multicenter randomized controlled trial evaluating a high-fidelity simulation-based training program for the management of traumatic hemorrhage, Starosolski et al. (2024) reported significant improvements in objective performance indicators related to bleeding control. These findings suggest that increases in perceived competence following simulation-based training may translate into measurable improvements in clinical performance [26]. Klein et al. (2025) further demonstrated that simulation training in hospital-based resuscitation scenarios was associated with improved self-efficacy among professional caregivers [27]. In addition, a high-fidelity telesimulation study by Peng et al. (2024) reported significant gains in emergency and critical care knowledge, self-confidence, and critical thinking skills compared with a control group [28].

The increase in students’ perceived competence observed in our study indicates that simulation-based education is effective not only in enhancing cognitive outcomes but also in promoting perceptual and affective gains. This improvement appears to reinforce the relationship between practice frequency and self-efficacy. Accordingly, gains were observed in both technical performance and learner confidence (Table 6). However, it should be noted that the correlations between practice frequency and perceived competence were weak to moderate, indicating an associative relationship rather than a strong predictive or causal effect. Consistent with these findings, Ertem et al. (2025) reported that a simulation study designed to realistically model the prehospital setting resulted in significant increases in students’ self-efficacy scores [29]. Similarly, Ohira et al. (2024) demonstrated that high-fidelity simulation-based focused ultrasound training improved the clinical performance of emergency medical technicians by reducing procedure times and increasing scores on both task-specific checklists and global rating scales [6]. These results are in line with the systematic review by Hegland et al. (2017), which showed that simulation-based training significantly enhanced nurses’ knowledge and skills, with particularly strong effects on self-efficacy, while also highlighting the role of high-fidelity simulations in supporting clinical decision-making processes [13].

In our study, students’ perceived competence increased significantly following the training, as reflected in the post-training perceived competence scores (Table 6). In parallel, objective performance in the same clinical skills improved markedly, with substantial increases observed in both total performance scores and success rates across all assessed practical skills (Table 7). Notable gains were noted in advanced procedures such as endotracheal intubation, basic and advanced life support, defibrillation, and cardiac arrest management, indicating a meaningful enhancement in clinical skill performance. This finding aligns with evidence from nursing education, where high-fidelity simulation consistently enhances self-confidence and self-efficacy [11, 12]. Notable improvements in advanced skills such as endotracheal intubation mirror results from nursing education, where targeted simulation programs have successfully increased competency and confidence in airway procedures [10]. A similar relationship was reported by Platt et al. (2024), where three consecutive simulation sessions led to significant gains in knowledge and self-efficacy among undergraduate students [30]. Likewise, a randomized controlled study found that VR-supported CPR training enhanced both the quality of skills and students’ confidence [31]. Another study with nursing students demonstrated that repeated ACLS simulations using the Rapid Cycle Deliberate Practice (RCDP) model were more effective than conventional simulation methods in improving knowledge, practical skills, and team communication [32]. A multicenter cross-sectional analysis (*n* = 412) also showed that active learning components within high-fidelity simulations were positively correlated with student satisfaction and self-confidence [33]. In line with these findings, a field-based trauma simulation study conducted among paramedic students reported significant improvements in technical skills and self-efficacy, reinforcing both performance and confidence [29]. Consistent with evidence from nursing education [9, 11], our findings support the structured integration of high-fidelity simulation into paramedic training programs.

From an educational perspective, the findings of this study have several important implications for paramedic education. The observed improvements in clinical skill performance and perceived competence support the integration of structured, simulation-based training as a core component of paramedic curricula rather than as a supplementary learning activity. In addition, these results highlight the importance of ongoing faculty development to ensure that instructors are adequately trained in simulation facilitation, debriefing strategies, and the use of standardized assessment tools. Finally, incorporating simulation-based assessment methods, such as OSCEs and structured skill checklists, may enhance the objectivity and practice-oriented evaluation of clinical competence in paramedic education programs. In addition, to avoid overgeneralization, it should be taken into account that the study sample consisted predominantly of female participants (83.3%) and was drawn from a single region. In this context, supporting these findings with studies conducted in different demographic groups and educational settings may further strengthen the transferability of the results.

### Limitations

This study has several limitations. First, the use of a one-group pretest–posttest design without a control group and randomization limits the ability to draw causal inferences regarding the effectiveness of the simulation-based intervention. In addition, the study was conducted with students enrolled in the First and Emergency Aid Program of a single public university, which restricts the generalizability of the findings to other institutions or educational contexts.

The single-center design and the relatively small sample size may further limit the external validity of the results. Clinical skill assessments were performed by the same instructors in both the pre- and post-test phases, and blinding was not applied. Although standardized scoring criteria were used, the potential for observer bias or a halo effect cannot be completely excluded and should be considered when interpreting the findings. Moreover, perceived competence and practice frequency were assessed using self-reported measures, which may be subject to response bias or social desirability effects. In addition, as multiple clinical skills were analyzed separately, the potential for an increased risk of Type I error should be considered when interpreting the results.

Finally, the effects of simulation- and practice-based training were evaluated only in the short term, and it remains unclear to what extent these improvements are sustained over time and transferred into professional competence after graduation. Notably, no loss to follow-up occurred, and all 60 students completed both the pre- and post-test assessments. Therefore, future multicenter studies with control groups, randomization, and longitudinal designs are recommended to confirm the long-term impact of simulation-based education on paramedic training.

## Conclusions

This study demonstrated that practice-based and simulation-supported training effectively enhanced the clinical skills, perceived competence, and confidence of paramedic students. Prior to training, most students had little or no experience with critical interventions and reported low levels of self-perceived competence. Following training, significant improvements were observed in both procedural success and confidence, with high achievement particularly in basic life support, intravenous access, oxygen administration, and fracture stabilization. A positive correlation between practice frequency and perceived competence suggests that hands-on experience plays a key role in building both skills and self-efficacy.

Overall, these results highlight that practice- and simulation-focused educational models are effective in supporting holistic development across cognitive, psychomotor, and affective domains. They suggest that structured simulation programs should be further integrated into paramedic curricula to strengthen professional readiness and ensure better preparedness for real-world emergencies.

## Data Availability

The data supporting the findings of this study are summarized in the article. Further details or raw data may be made available by the authors upon reasonable request.
